# Factors Associated with COVID-19 Vaccine Hesitancy after Implementation of a Mass Vaccination Campaign

**DOI:** 10.3390/vaccines10020281

**Published:** 2022-02-12

**Authors:** Inês Afonso Gomes, Patricia Soares, João Victor Rocha, Ana Gama, Pedro Almeida Laires, Marta Moniz, Ana Rita Pedro, Sónia Dias, Ana Rita Goes, Andreia Leite, Carla Nunes

**Affiliations:** 1 Comprehensive Health Research Center, Universidade NOVA de Lisboa, Campo Mártires da Pátria 130, 1169-056 Lisboa, Portugal; patseraos@gmail.com (P.S.); jv.rocha@ensp.unl.pt (J.V.R.); ana.gama@ensp.unl.pt (A.G.); laires.pedro@gmail.com (P.A.L.); am.moniz@ensp.unl.pt (M.M.); rita.pedro@ensp.unl.pt (A.R.P.); sonia.dias@ensp.unl.pt (S.D.); ana.goes@ensp.unl.pt (A.R.G.); andreia.leite@ensp.unl.pt (A.L.); cnunes@ensp.unl.pt (C.N.); 2 NOVA National School of Public Health, Public Health Research Center, Universidade NOVA de Lisboa, Av. Padre Cruz, 1600-560 Lisboa, Portugal; 3 Laboratórios Pfizer Lda., Lagoas Park, 2740-271 Porto Salvo, Portugal

**Keywords:** COVID-19, vaccination, vaccine hesitancy

## Abstract

An online cross-sectional study on COVID-19 vaccination adhesion was conducted in Portugal nine months after vaccination rollout (September–November 2021). Logistic regression was used to identify factors associated with hesitancy to take the COVID-19 vaccine in the community-based survey, “COVID-19 Barometer: Social Opinion”. Hesitancy was 11%; however, of those, 60.5% stated that they intended to take the vaccine. Hesitancy was associated with factors such as lower monthly household income; no intention of taking the flu vaccine this year; perceived reasonable health status; having two or more diseases; low confidence in the health service response; worse perception of the adequacy of anti-COVID-19 government measures; low or no perceived risk of getting COVID-19; feeling agitated, anxious or sad some days; and lack of trust in the safety and efficacy of the vaccines. Confidence in vaccines, namely against COVID-19, is paramount for public health and should be monitored during vaccination rollout. Clear communication of the risks and benefits of vaccination needs improvement to increase adherence and public confidence.

## 1. Introduction

Until 22 November 2021, more than 262 million COVID-19 cases and 5.2 million COVID-19 deaths had been confirmed worldwide [1]. The availability of vaccines has brought a new breath of hope for a return to “normal life” [2]. However, with this accelerated vaccine development, the issue of vaccine hesitancy has resurfaced [3,4,5,6], leveraging both scientific and public debate on this topic and its impact on the success of immunisation programs [3,4,5,6,7,8,9,10]. The European Union vaccination targeted a 70% vaccination rate of the adult population by the summer of 2021, reaching 71.6% on 12 September 2021 [2]. However, pockets of low vaccine uptake, which can compromise the vaccination coverage, still exist in this region [2]. Hence, the risk of increasing numbers of severe cases, hospitalisations and deaths associated with this disease still represent a major public health concern [2].

Vaccine hesitancy was identified by the World Health Organization (WHO) as one of the top 10 threats to global health in 2019 [11], and it can be defined as the delay in acceptance, reluctance, or refusal of vaccination despite the availability of vaccination services [11,12]. Vaccine hesitancy is the outcome of a complex decision-making process [2,12], dependent on multiple factors [8,9,10,12], namely contextual, individual, group, and vaccine-specific factors [12].

As the pandemic progressed, people reporting their intention to be vaccinated against COVID-19 varied [4,6,7,13,14,15,16,17,18,19]. However, most studies on COVID-19 vaccine acceptance were conducted before the first vaccine’s approval or vaccination program rollout. Hence, the reported vaccine acceptance or hesitancy was mostly assessed using hypothetical questions as there were still no vaccines available outside of the clinical trial setting [3,4,5,6,8,9,10]. Several cross-sectional studies have since explored COVID-19 vaccine acceptance and hesitancy and their determinants after the respective countries’ vaccination program implementation, namely in European countries [2], such as the United Kingdom [15,16,20,21], Germany [15,22], Denmark [15,23], France [15], Hungary [15], Sweden [15], Italy [15], Norway [21], Poland [24], the Netherlands [25], Spain [26] and Greece [27]. Additional studies were also conducted in Saudi Arabia [28], the United States of America [13,17,21,29], South Africa [21,30], Jordan [31], Israel [32], Chile [33], China [18,21,34,35], Japan [21,36] and Australia [21], among others [21]. These studies have not only reported variation in the refusal rate of COVID-19 vaccination over sequential waves [13,14,15,18], but also on the characteristics of the individuals that reported hesitancy in getting vaccinated [13,14,18] and on the reasons behind this refusal [14].

In these studies, COVID-19 vaccination acceptance or hesitancy varied according to age [2,3,4,6,8,9,10,13,14,16,17,18,20,21,22,24,30,32,33,34,35,36,37,38], gender [3,6,10,13,14,16,18,20,21,22,24,26,28,30,31,36], education [4,6,8,9,10,13,14,17,18,21,22,24,27,28,34,36,38,39], race/ethnicity [2,13,14,16,20,35,37], political influence [13,15,24], religion [2,37], profession [2,17,18,19,20,27,31,32], employment status [24,30], socioeconomic status [2,4,5,6,8,10,14,16,17,18,20,34,36] and place of residence [2,14,15,17,18,24,30,34,39]. The main source of information on the COVID-19 vaccine [22,27,28,29,31], belief in COVID-19 vaccination’s effectiveness against new variants [28,31,37] and requirement for vaccination against this virus for international travelling [28] were also described as being associated with COVID-19 vaccination acceptance/hesitancy. Additionally, prior vaccination against the flu [5,8,9,10,18,27,28,38], anxiety/depression symptoms [18], self-perception of poor health [38] or presence of comorbidities [8,18,38] were associated with acceptance of COVID-19 vaccines.

Negative perceptions on the safety [5,8,10,13,14,16,17,18,19,21,25,27,29,30,31,33,37] or efficacy/effectiveness [5,8,13,14,16,17,19,21,25,31,34,37] of these vaccines and misinformation on this topic [2,15] were described in individuals hesitant about COVID-19 vaccination. Additionally, a perception of COVID-19 as not a risk to self [5,8,10,14,15,16,17,18,22,25,27,30,31,32,33,37,38], as well as general distrust of COVID-19 vaccines [14,17], the government [14,15,19,23,30] or a lack of trust in the vaccines’ development [13,14,19,30,32] and approval process [14,15,19] were reported in hesitant individuals. Additionally, media reports on the safety and efficacy of specific vaccine brands led to a reduction in trust in the safety of those vaccines [23,30] and the vaccination process [30]. Conspiracy beliefs [15,19] and lack of concerns on health system constraints [15] were also linked to hesitancy towards these vaccines. Furthermore, the use of new technologies, such as mRNA [21,27,37], as well as the mass use of conditionally approved medicines were pointed out as factors that could increase COVID-19 vaccine hesitancy [37].

Portugal has one of the highest COVID-19 vaccination coverages worldwide [7], with 88.8% of the eligible population being fully vaccinated by 13 December 2021 [40]. This vaccination rate is higher than the global and European Union rates (46.3% and 68.1%, respectively) [40]. In a study conducted among Portuguese immunosuppressed cancer patients three months after implementing the vaccination program, the vaccine acceptance was 84.0% [39]. However, in a prior cross-sectional analysis of the Portuguese community-based survey “COVID-19 Barometer: Social Opinion” using data from 29 September 2020 to 8 January 2021, only 35.3% of the individuals would like to be vaccinated as soon as possible, with most (55.5%) preferring to wait some time before being vaccinated and 9.2% refusing the vaccination [38]. These data suggest variations in the vaccine acceptance rate over time, which deserves further examination and continuous monitoring.

Additionally, the world has been facing the emergence of new virus mutations with potential higher transmissibility and virulence [41] and new threats to immunity, including waning and new variants [42]. By the end of 2021, many countries have experienced new waves of the COVID-19 pandemic, and on 4 November the World Health Organization declared, once again, Europe as the epicentre of the pandemic [43]. Thus, understanding the reasons behind vaccine hesitancy [2], specifically throughout the vaccination campaign rollout, is of paramount importance to tailor policies and media campaigns to guarantee a high coverage for these new vaccines [3]. Hence, this study aims to assess and identify factors associated with COVID-19 vaccine hesitancy in Portugal, nine months after the rollout of the country’s vaccination program, in a context of high vaccination coverage.

## 2. Materials and Methods

### 2.1. Study Design

We used data from the community-based survey “COVID-19 Barometer: Social Opinion”, launched by the National School of Public Health at the NOVA University of Lisbon (ENSP/NOVA), to identify and monitor the perception of the population regarding the pandemic and its impact on their health, wellbeing and daily-life [44]. The ongoing questionnaire was implemented at the beginning of the COVID-19 pandemic in 2020, with more than 220,000 answers by 30 November 2021. The questionnaire is flexible and allows for the inclusion of new questions and withdrawal of old ones according to the pandemic context. We analysed data between 18 September and 26 November 2021, approximately nine months after the country’s vaccination program began. This time frame was selected because, according to the country’s vaccination strategy and availability, every citizen older than 16 years old willing to be vaccinated would have access to it and could be vaccinated before our study started. By 20 September, 84.1% of the eligible population was fully vaccinated, and that number increased to 88.4% by 29 November [40]. Participants who were not living in Portugal were excluded from the analysis. Participants could fill in the questionnaire once or every two weeks, which would allow the analysis of trends and variations in responses [44]. We only used one questionnaire per participant for this study and considered the last time the participant answered the questionnaire between 18 September and 26 November 2021.

### 2.2. Outcome

The outcome question was “What is your intention to get the COVID-19 vaccine when called?”. In this question, participants could answer whether they wanted to take the vaccine, were undecided, did not want to take it, or were already vaccinated. For the analysis, the dependent variable was categorised into vaccinated and hesitant. Participants who answered “I am already vaccinated” corresponded to vaccinated individuals. In contrast, the remaining participants, i.e., participants who answered “I will take it”, “I have not decided yet” and “No” were considered hesitant as all these answers represented some form of delay, reluctance or vaccination refusal [12].

### 2.3. Independent Variables

The independent variables collected by the survey “COVID-19 Barometer: Social Opinion” and included in this analysis were those considered potentially associated with vaccine hesitancy. These variables were grouped following the Working Group on Vaccine Hesitancy framework as contextual, individual and group, and vaccine-specific influences [12], with a fourth category added regarding disease-specific variables. This classification was used in a prior cross-sectional analysis in Portugal using data from before the vaccines were widely available for the population [38]. Table 1 presents the variables considered for this analysis and their categories.

### 2.4. Statistical Analysis

The study outcome (dependent variable) was binary, vaccinated or hesitant, and the independent variables were grouped based on the vaccine hesitancy determinant matrix in Table 1. This self-reported vaccination rate corresponded to the primo-vaccination rate, as at this time, the booster campaign had not yet been extended to the general population. Variables were initially described using absolute and relative frequencies. Logistic regression models were fitted. We estimated a crude odds-ratio (OR) for all variables with the corresponding 95% confidence interval (95% CI). Our interest was to assess and identify factors associated with COVID-19 vaccine hesitancy, an explanatory aim and not predictive, and we adjusted separate logistic regression, adjusted for age, gender, education, and month of the questionnaire, for each variable of interest.

We conducted a sensitivity analysis without participants who answered “I will take the COVID-19 vaccine”. We assumed that those participants might be less hesitant than those who answered that they would delay or refuse to take the vaccine.

All statistical analyses were performed using R 4.0.2 [45].

## 3. Results

A total of 3232 individuals were included in the analysis, of which 2875 (89%) were vaccinated and the remaining 375 (11%) were hesitant. The distribution of hesitant consisted mainly of participants who answered “I will take the vaccine” (60.5%), followed by participants who said they would not take the vaccine (27.5%) and undecided participants (12%). The characteristics of the sample are presented in Table 2, Table 3, Table 4 and Table 5.

Most of the individuals that answered the questionnaire were women (75%), aged between 25 and 64 years (69%) and had a university degree (76%). Around 55% of those hesitant about the COVID-19 vaccine perceive their health status as very good/good. The majority of vaccinated participants (90%) perceived COVID-19 vaccines as safe and effective, while the proportion of hesitant participants who perceived the COVID-19 vaccines as safe and effective was around 64%.

The results of the regression models are presented according to the groups on the vaccine hesitancy determinant matrix. The OR and aOR and their respective 95% CI are in the Supplementary Materials (Table S1). The sensitivity analysis can also be found in the Supplementary Materials (Table S2).

### 3.1. Determinants of Vaccine Hesitancy: Contextual Influences

**Figure 1:**
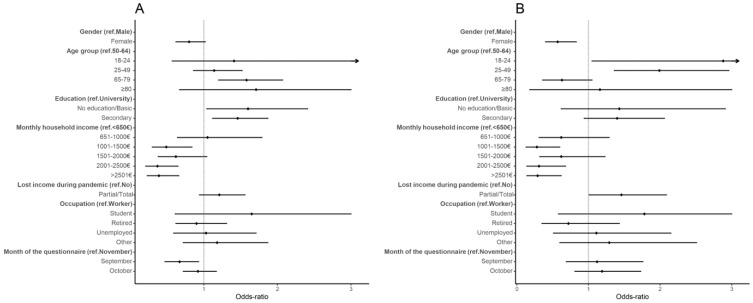
Forest plot of vaccine hesitancy for contextual influences. Adjusted odds ratio (adjusted for gender, age, education, and period of questionnaire) and the respective 95% confidence intervals are denoted by black dots and black lines, respectively. Forest plot confidence intervals were cut off at 3. (**A**) Results of the main analysis—vaccinated vs. hesitant (refuse, undecided and would take the vaccine); (**B**) results for the sensitivity analysis, removing individuals who would take the vaccine.

Participants aged 65 to 79 years had higher odds of hesitancy than participants aged 50 to 64 years (aOR: 1.58, 95% CI: 1.20, 2.07). Higher odds of vaccine hesitancy were also found for individuals with no education/basic education and secondary education, when compared to those with a university degree (no education/basic education aOR: 1.60, 95% CI: 1.04, 2.41; secondary aOR: 1.46, 95% CI: 1.12, 1.87). Participants with higher monthly household incomes had lower odds of hesitancy than participants with a monthly household income smaller than EUR 650. Participants who answered the questionnaire in September had lower hesitancy odds than those who answered in November (aOR: 0.67, 95% CI: 0.47, 0.93) (Table S1, Figure 1).

In the sensitivity analysis, i.e., excluding individuals who answered that they would take the vaccine, education was no longer associated with vaccine hesitancy. In turn, gender and loss of income became statistically significant, with women presenting lower odds of hesitancy (aOR: 0.57, 95% CI: 0.40, 0.83), and participants who lost income during the pandemic presenting higher odds of hesitancy (aOR: 1.46, 95% CI 1.01, 2.09). Interestingly, the results for age changed in the sensitivity analysis, with participants aged less than 50 years presenting higher odds of being hesitant than participants between 50 and 64 years old (16–24 years aOR: 2.88, 95% CI 1.05, 6.73; 25–49 aOR: 1.99, 95% CI: 1.36, 2.96). The association with monthly household income was still present (Table S2, Figure 1).

### 3.2. Determinants of Vaccine Hesitancy: Individual Influences

**Figure 2:**
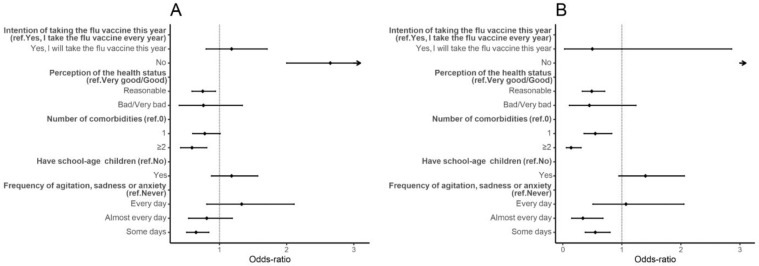
Forest plot of vaccine hesitancy for individual influences. Adjusted odds-ratio (adjusted for gender, age, education, and period of questionnaire) and the respective 95% confidence intervals are denoted by black dots and black lines, respectively. Forest plot confidence intervals and estimates were cut off at 3. (**A**) Results of the main analysis—vaccinated vs. hesitant (refuse, undecided and would take the vaccine); (**B**) results for the sensitivity analysis, removing individuals who would take the vaccine.

Individuals who did not intend to take the flu vaccine this year had higher hesitancy odds than those who took or intended to take the flu vaccine (aOR: 2.65, 95% CI: 2.00, 3.53). Those who perceived their health status as reasonable had lower odds of hesitancy than those who perceived it as very good/good (aOR: 0.75, 95% CI: 0.59, 0.94). Participants who had two or more diseases also had lower odds of hesitancy than participants without diseases (aOR: 0.59, 95% CI: 0.42, 0.81). Participants who reported feeling agitated, sad, or anxious some days due to the physical distance measures had lower odds of hesitancy than participants who reported never having these feelings (aOR: 0.65, 95% CI: 0.51, 0.84) (Table S1, Figure 2). Results were similar in the sensitivity analysis (Table S2, Figure 2).

### 3.3. Determinants of Vaccine Hesitancy: COVID-19 Influences

**Figure 3:**
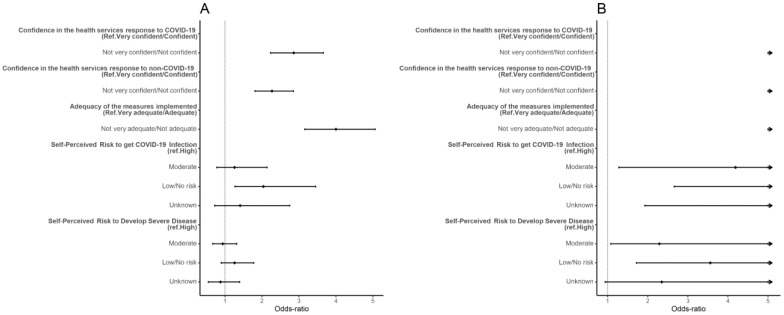
Forest plot of vaccine hesitancy for COVID-19 influences. Adjusted odds ratio (adjusted for gender, age, education, and period of questionnaire) and the respective 95% confidence intervals are denoted by black dots and black lines, respectively. Forest plot confidence intervals and estimates were cut off at 5. (**A**) Results of the main analysis—vaccinated vs. hesitant (refuse, undecided and would take the vaccine); (**B**) results for the sensitivity analysis, removing individuals who would take the vaccine.

Increased odds of vaccine hesitancy were found for participants that had low or no confidence in the health services’ response to COVID-19 and non-COVID-19 needs (COVID-19 aOR: 2.86, 95% CI: 2.23, 3.36; non-COVID-19 aOR: 2.27, 95% CI: 1.81, 2.85), and participants who found the measures implemented by the government to be inadequate (aOR: 4.00, 95% CI: 3.16, 5.06). Participants who perceived low or non-existent risk of getting COVID-19 infection had higher odds of hesitancy than participants who perceived their risk as high (aOR: 2.04, 95% CI: 1.27, 3.45) (Table S1, Figure 3).

Results were similar in the sensitivity analysis. Increased odds of hesitancy were found for participants who had low confidence in the health services’ response to COVID-19 and non-COVID-19 and who perceived the measures implemented by the government as inadequate. Participants who perceived high risk of getting COVID-19 and developing severe disease had lower odds of hesitancy (Table S2, Figure 3).

### 3.4. Determinants of Vaccine Hesitancy: COVID-19-Vaccine-Related Influences

Participants who perceived the COVID-19 vaccines as unsafe had higher hesitancy odds than those who perceived them as safe (aOR: 15.82, 95% CI 11.67, 21.54). Similarly, participants who perceived the COVID-19 vaccines as ineffective had higher hesitancy odds than those who perceived them as effective (aOR: 10.32, 95% CI 7.81, 13.64) (Table S1). In the sensitivity analysis, results for the safety and efficacy of the COVID-19 vaccines were similar, but the association with time was no longer significant (Table S2).

## 4. Discussion

The present study investigated the factors associated with vaccine hesitancy in 3232 participants from the “COVID-19 Barometer: Social Opinion”, nine months after the vaccination rollout in Portugal, following completion of the mass vaccination campaign among people aged 16 years and older. Most participants were already vaccinated (89%) at the time of the study. This self-reported vaccination rate corresponded to the primo-vaccination rate, as, at this time, the booster campaign had not yet been extended to the general population. We found that the following factors were associated with hesitancy in the main and sensitivity analysis: (i) contextual factors: lower monthly household income; (ii) individual factors: no intention of taking the flu vaccine this year, perceiving their health status as reasonable and having two or more diseases; (iii) COVID-19 influences: low confidence in the health service response to COVID-19 and non-COVID-19, worse perception of the adequacy of measures implemented by the government, perceiving a low or non-existent risk of getting COVID-19, and feeling agitated, anxious or sad some days due to the physical distance measures; and (iv) COVID-19-vaccine-specific factors: lack of trust in the safety and efficacy of the vaccines.

We found that 11% of the participants were still hesitant in a highly adhering setting. To the best of our knowledge, this is the first estimate following completion of the mass vaccination in individuals aged 16 and above. By 14 November 2021, official reports reported a 100% vaccination rate for individuals older than 64, 99% for individuals between 50 and 64, 94% for individuals between 25 and 49, 91% for individuals between 18 and 24 and 87% for individuals between 12 and 17 years old [46]. Our results and further data on un- or incompletely vaccinated hospitalised individuals [47] indicate that existing vaccination coverage is likely overestimated. Yet, 60.5% of those hesitant corresponded to participants who stated that they would take the vaccine, thus representing a further opportunity for public health authorities and professionals to invite individuals to get vaccinated. It is crucial to explore further why some individuals are keen to take the vaccine but have not done it yet at the time of the study. The safety and efficacy perception in the vaccines among the vaccinated group were 96% and 94%, respectively. Relatively high values were also found among the hesitant group (65% and 63%, respectively), which may indicate that, despite perceiving vaccines as safe and effective, other factors in the COVID-19 disease-specific category may make people hesitant (e.g., less fear for health or worry about COVID-19, perception of lower risk of infection, belief that it is not a severe disease). These factors have been explored in the literature [48], and further quantitative and qualitative studies can shed some light on possible reasons for this phenomenon and potentially reduce hesitancy by tackling some causes behind no vaccination for these individuals. Additional research could also further explore whether this delay is related to fears associated with vaccination or individual health status, or due to practical reasons, such as difficult access, inability to leave work, among others.

On the other hand, hesitancy has decreased since the beginning of the vaccination programme. At the beginning of the year, we found a hesitancy rate of 65% using data from the same survey [38]. This difference indicates the complexity of vaccine hesitancy and how hesitancy changes over time as more information is made available. Our previous study found that questionnaires answered before the release of information regarding the safety and efficacy of COVID-19 vaccines were strongly associated with higher odds of hesitancy [38]. These results obtained using the same questionnaire at two very different time points highlight the need to assess vaccination intent and uptake [3] once a vaccination program is rolled out and over time. Hesitancy rates and the characteristics of hesitant subjects might vary, and monitoring of hesitancy rates was previously recommended by the ECDC [2]. This is particularly relevant as booster doses are currently being administered [49] with a lower uptake than previously observed for the primo vaccination.

Negative perceptions towards the efficacy [5,8,13,14,17,19,21,25,31,34,37] and safety [5,8,10,13,14,16,17,18,19,21,25,27,29,30,31,33,37,40] of COVID-19 vaccines were highly linked to vaccine hesitancy. Given the complexity of this topic, aligned with the growing misinformation present in several media regarding the development process of vaccines, concerns about the safety and efficacy of the vaccines are understandable [2,15]. Additionally, the population’s level of trust in vaccines and the availability of understandable and reliable information on these uncertain times can be impacting factors in the adherence to this vaccination [50]. There is a need to implement strategies to address the population perception and misconceptions regarding the efficacy and safety of COVID-19 vaccines, as these are strongly associated with hesitancy. Health status and having diseases were other important factors in vaccine hesitancy. This study found that participants who perceived their health status as reasonable and had two or more diseases had lower odds of hesitancy than participants with a good health perception and no diseases [8,18,34,36,40,46]. Additionally, we found that participants who felt negative emotions some days had lower odds of hesitancy.

We also found higher odds of hesitancy for participants with low confidence in the health services response to COVID-19 and non-COVID-19 and who perceived the measures implemented by the government as inadequate, matching prior publications on this topic [15,46]. Higher odds of hesitancy were also found for participants who perceived their risk of getting COVID-19 as low or non-existent, which was in agreement with the literature [5,8,10,14,15,16,17,18,22,25,27,30,31,33,37,46]. Risk perception and trust in the authorities and health institutions can vary according to a country’s pandemic context during a specific period [51]. For instances, the low infection rate during the period of analysis (with a 7-day rolling average ranging from 94.11 to 266.88 per million inhabitants on 18 September and 26 November, respectively [40]) could be detrimental to the vaccination drive in this particular time period.

Furthermore, no intention of taking the flu vaccine in the current year was associated with hesitancy to the COVID-19 vaccination, matching prior data on this topic [5,8,18,27,28,46] and suggesting that global attitudes towards these vaccines may cluster together to influence decision making.

Lower odds of hesitancy were found for participants with higher monthly household incomes [2,4,5,6,8,10,14,16,17,20,34]. We also found lower odds of hesitancy for individuals with a university degree, which is in agreement with other studies [4,6,8,9,10,13,14,17,18,21,22,24,27,28,34,46]. However, analysing only participants that refused or were undecided, this association is no longer significant. Loss of income during the pandemic was described as a hesitancy driver in the sensitivity analysis. This association aligned with the outcomes of the prior analysis of this survey conducted at the beginning of the year [46]. It is important to further explore these results in different socioeconomic groups with different educations, as vaccine hesitancy determinants do not have associations in only one direction [12]. For instance, our previous results showed that higher household income was associated with lower odds of hesitancy for individuals without a university degree. In contrast, higher household income was associated with higher odds of hesitancy for individuals with a university degree [38].

We found divergent results in the main and sensitivity analyses regarding age. Considering all participants who reported not being vaccinated, higher odds of hesitancy were found for individuals aged between 65 and 79 years old than individuals between 50 and 64 years old. However, removing from the analyses participants who reported intention to vaccinate, we found that participants younger than 49 had higher odds of hesitancy than individuals between 50 and 64 years old. The literature is in agreement with higher hesitancy for younger age groups [2,3,4,6,8,9,10,13,14,16,17,18,20,22,24,30,33,36,37,40,46]. We categorised age using the same age groups used by the official Portuguese reports for our analysis, which indicate a similar trend [46]. Other results lost or gained statistical significance in the sensitivity analysis. Women had lower odds of hesitancy only in the sensitivity analysis, although in the main analysis, the upper limit of the confidence interval was 1.02. In contrast, our previous study found increased odds of delaying vaccination for women [38], suggesting once more the changes associated with hesitancy over time. However, several studies reported lower odds of hesitancy for women [21], although this result was not always replicated [3,6,10,13,18,20,24,26,28]. When considering the month of response, the lower hesitancy for participants answering the questionnaire in September (versus November responders) lost statistical significance during the sensitivity analysis. Nevertheless, this result points to a temporal variation in vaccination hesitancy, as previously reported by other authors [13,14,15,18], highlighting the need for continuous monitoring.

One of the strengths of the present study is the collection of data through a nationwide survey, analysed using a comprehensive framework on vaccine hesitancy that allowed us to identify contextual, individual, and vaccine-specific factors associated with vaccine uptake and hesitancy. This study was based on an online, voluntary survey and presented some limitations. The sample is not representative of the Portuguese population, as there was a clear preponderance of women and participants with higher education levels. There is a possibility of sampling bias (due to the online nature of the survey), selection bias (participants might be more conscious of the seriousness of COVID-19 than non-participants), and response, non-response and social desirability bias. Despite these limitations, our study indicates that official data on coverage are likely overestimated.

In conclusion, Portuguese adults showed good adherence to the COVID-19 mass vaccination efforts, with hesitancy remaining in a sub-group mostly due to delayed vaccination. Negative vaccine-related perceptions, low risk perceptions and general sentiments of distrust were identified as important factors associated with hesitancy. These factors can inform the development of tailored interventions to tackle COVID-19 vaccine hesitancy.

## Figures and Tables

**Table 1:** Vaccine hesitancy determinant matrix recommended by the Strategic Advisory Group of Experts (SAGE) Working Group on Vaccine Hesitancy [12], with the fourth category specific to the COVID-19 disease [38].

Determinants of Vaccine Hesitancy	Variables
Contextual influences	Gender
	Age group
	Education
	Monthly household income
	Partial or total income loss during the pandemic
	Occupation Month of the questionnaire
Individual influences	Intention to take the flu vaccine
	Perception of the health status
	Number of comorbidities
	Having school-age children Frequency of agitation, sadness, or anxiety
COVID-19 disease-specific	Confidence in the health services response to COVID-19
	Confidence in the health services response to non-COVID-19
	Perception of the adequacy of measures implemented by the Government
	Self-perceived risk of getting COVID-19 infection
	Self-perceived risk of developing severe disease following COVID-19 infection
COVID-19 vaccine-specific	Confidence in the safety of the COVID-19 vaccines
	Confidence in the efficacy of the COVID-19 vaccines

**Table 2:** Sample characteristics according to contextual influences.

Contextual Influences	Vaccinated (N = 2857)	Hesitant (N = 375)	Total (N = 3232)
Gender (N = 3224)			
Male	686 (24.1%)	117 (31.3%)	803 (24.9%)
Female	2164 (75.9%)	257 (68.7%)	2421 (75.1%)
Age group (N = 3232)			
16–24	44 (1.5%)	7 (1.9%)	51 (1.6%)
25–49	933 (32.7%)	106 (28.3%)	1039 (32.1%)
50–64	1080 (37.8%)	117 (31.2%)	1197 (37.0%)
65–79	767 (26.8%)	138 (36.8%)	905 (28.0%)
80+	33 (1.2%)	7 (1.9%)	40 (1.2%)
Education (N = 3221)			
No education/Basic education	149 (5.2%)	32 (8.6%)	181 (5.6%)
Secondary	609 (21.4%)	103 (27.7%)	712 (22.1%)
University	2091 (73.4%)	237 (63.7%)	2328 (72.3%)
Monthly household income (N = 2958)			
<EUR 650	118 (4.5%)	26 (8.1%)	144 (4.9%)
EUR 651–1000	275 (10.4%)	60 (18.8%)	335 (11.3%)
EUR 1001–1500	523 (19.8%)	57 (17.8%)	580 (19.6%)
EUR 1501–2000	509 (19.3%)	72 (22.5%)	581 (19.6%)
EUR 2001–2500	474 (18.0%)	39 (12.2%)	513 (17.3%)
>EUR 2501	739 (28.0%)	66 (20.6%)	805 (27.2%)
Lost of income due to the pandemic (N = 3165)			
No	2082 (74.4%)	264 (72.3%)	2346 (74.1%)
Partial/Total	718 (25.6%)	101 (27.7%)	819 (25.9%)
Occupation (N = 3232)			
Worker	1736 (60.8%)	194 (51.7%)	1930 (59.7%)
Student	52 (1.8%)	10 (2.7%)	62 (1.9%)
Retired	764 (26.7%)	127 (33.9%)	891 (27.6%)
Unemployed	143 (5.01%)	17 (4.53%)	160 (4.95%)
Other	162 (5.67%)	27 (7.20%)	189 (5.9%)
Month (N = 3232)			
September	454 (15.9%)	45 (12.0%)	499 (15.4%)
October	848 (29.7%)	110 (29.3%)	958 (29.6%)
November	1555 (54.4%)	220 (58.7%)	1775 (54.9%)

**Table 3:** Sample characteristics according to individual influences.

Individual Influences	Vaccinated (N = 2857)	Hesitant (N = 375)	Total (N = 3232)
Intention of taking the flu vaccine this year (N = 3178)			
Yes, I take the flu vaccine every year	1224 (43.6%)	126 (34.2%)	1350 (42.5%)
Yes, I will take the flu vaccine this year	428 (15.2%)	42 (11.4%)	470 (14.8%)
No	1158 (41.2%)	200 (54.3%)	1358 (42.7%)
Perception of the health status (N = 3226)			
Very good/Good	1421 (49.8%)	205 (55.1%)	1626 (50.4%)
Reasonable	1326 (46.5%)	154 (41.4%)	1480 (45.9%)
Bad/Very bad	107 (3.75%)	13 (3.49%)	120 (3.72%)
Number of diseases (N = 3160)			
0	1339 (47.8%)	188 (52.2%)	1527 (48.3%)
1	866 (30.9%)	111 (30.8%)	977 (30.9%)
≥2	595 (21.2%)	61 (16.9%)	656 (20.8%)
Have school-age children (N = 3222)			
No	2042 (71.7%)	274 (73.5%)	2316 (71.9%)
Yes	807 (28.3%)	99 (26.5%)	906 (28.1%)
Frequency of agitation, sadness or anxiety (N = 3218)			
Never	772 (27.1%)	135 (36.1%)	907 (28.2%)
Some days	1655 (58.2%)	176 (47.1%)	1831 (56.9%)
Almost every day	291 (10.2%)	38 (10.2%)	329 (10.2%)
Every day	126 (4.4%)	25 (6.7%)	151 (4.7%)

**Table 4:** Sample characteristics according to COVID-19 influences.

COVID-19 Influences	Vaccinated (N = 2857)	Hesitant (N = 375)	Total (N = 3232)
Confidence in the health services response to COVID-19 (N = 3213)			
Very confident/Confident	2416 (84.8%)	245 (67.1%)	2661 (82.8%)
Not very confident/Not confident	432 (15.2%)	120 (32.9%)	552 (17.2%)
Confidence in the health services response to non-COVID-19 (N = 3210)			
Very confident/Confident	1623 (57.2%)	140 (37.7%)	1763 (54.9%)
Not very confident/Not confident	1216 (42.8%)	231 (62.3%)	1447 (45.1%)
Perception of the adequacy of the measures implemented by the Government (N = 3172)			
Very adequate/Adequate	2220 (79.2%)	193 (52.3%)	2413 (76.1%)
Not very adequate/Not adequate	583 (20.8%)	176 (47.7%)	759 (23.9%)
Self-Perceived Risk of getting COVID-19 Infection (N = 3226)			
High	221 (7.8%)	19 (5.1%)	240 (7.4%)
Moderate	1321 (46.3%)	139 (37.1%)	1460 (45.3%)
Low/No risk	1143 (40.1%)	197 (52.5%)	1340 (41.5%)
Not sure	166 (5.8%)	20 (5.3%)	186 (5.8%)
Self-Perceived Risk of Developing Severe Disease Following COVID-19 Infection (N = 3221)			
High	415 (14.6%)	58 (15.5%)	473 (14.7%)
Moderate	1047 (36.8%)	124 (33.2%)	1171 (36.4%)
Low/No risk	1090 (38.3%)	159 (42.6%)	1249 (38.8%)
Not sure	296 (10.4%)	32 (8.6%)	328 (10.2%)

**Table 5:** Sample characteristics according to COVID-19-vaccine-related influences.

COVID-19 Vaccine-Related Influences	Vaccinated (N = 2857)	Hesitant (N = 375)	Total (N = 3232)
Safety perception in the COVID-19 vaccines (N = 3134)			
Completely safe/Safe	2670 (96.1%)	232 (65.0%)	2902 (92.6%)
Not very safe/Not safe	107 (3.9%)	125 (35.0%)	232 (7.4%)
Efficacy perception in the COVID-19 vaccines (N = 3143)			
Completely effective/Effective	2613 (93.9%)	228 (63.3%)	2841 (90.4%)
Not very effective/Not effective	170 (6.1%)	132 (36.7%)	302 (9.6%)

## Data Availability

The data presented in this study are available on request from the corresponding author. The data are not publicly available since this is an ongoing study.

## Supplementary Materials

The following are available online at https://www.mdpi.com/article/10.3390/vaccines10020281/s1, Table S1: Crude and adjusted odds of hesitancy for the determinants of vaccine hesitancy. Odds adjusted for gender, age, education and period of questionnaire; Table S2: Crude and adjusted odds of hesitancy, for participants who answered they would delay or refuse vaccination, for the determinants of vaccine hesitancy. Odds adjusted for gender, age, education and period of questionnaire.
