# Supplementary material for: Factors Associated with COVID-19 Vaccine Hesitancy after Implementation of a Mass Vaccination Campaign
Source: Vaccines (Basel). 2022 Feb 12;10(2):281. doi: 10.3390/vaccines10020281 (PMC8879669; doi:10.3390/vaccines10020281)
Supplement: Supplementary file 1 [file vaccines-10-00281-s001.zip › vaccines-1558190-supplementary.pdf]

Table S1: Crude and adjusted odds of hesitancy for the determinants of vaccine hesitancy. Odds adjusted for gender, age, education and period of questionnaire. OR: odds-ratio, CI: confidence interval.

	Crude		Adjusted	
	**OR**	**95% CI**	**OR**	**95% CI**
**Gender** (ref.Male)	**0.70**	**(0.55; 0.88)**	0.80	(0.62; 1.02)
**Age** (ref.50-64)				
16-24	1.47	(0.59; 3.14)	1.41	(0.57; 3.03)
25-49	1.05	(0.79; 1.38)	1.14	(0.86; 1.52)
65-79	**1.66**	**(1.28; 2.16)**	**1.58**	**(1.20; 2.07)**
80+	1.96	(0.78; 4.28)	1.71	(0.67; 3.77)
**Education** (ref.University)				
No education/Basic education	**1.89**	**(1.25; 2.81)**	**1.60**	**(1.04; 2.41)**
Secondary	**1.49**	**(1.16; 1.91)**	**1.46**	**(1.12; 1.87)**
**Monthly household income** (ref. <650€)				
651-1000€	0.99	(0.60; 1.67)	1.05	(0.64; 1.79)
1001-1500€	**0.49**	**(0.30; 0.83)**	**0.49**	**(0.30; 0.84)**
1501-2000€	0.64	(0.40; 1.06)	0.62	(0.38; 1.04)
2001-2500€	**0.37**	**(0.22; 0.64)**	**0.37**	**(0.21; 0.65)**
> 2501€	**0.41**	**(0.25; 0.67)**	**0.39**	**(0.23; 0.66)**
**Lost of income due to the pandemic** (ref. No)	1.11	(0.87; 1.41)	1.21	(0.94; 1.56)
**Occupation** (ref. Worker)				
Student	1.72	(0.81; 3.30)	1.65	(0.61; 3.99)
Unemployed	1.06	(0.61; 1.75)	1.03	(0.59; 1.71)
Retired	**1.49**	**(1.17; 1.89)**	0.90	(0.62; 1.31)
Other	1.49	(0.95; 2.27)	1.18	(0.72; 1.87)
**Month** (ref. November)				
September	**0.70**	**(0.49; 0.97)**	**0.67**	**(0.47; 0.93)**
October	0.92	(0.72; 1.17)	0.92	(0.72; 1.17)
**Intention of taking the flu vaccine this year** (ref. Yes, I take the flu vaccine every year)				
Yes, I will take the flu vaccine this year	0.95	(0.65; 1.36)	1.18	(0.80; 1.71)
No	**1.68**	**(1.33; 2.13)**	**2.65**	**(2.00; 3.53)**
**Perception of the health status** (ref. Very good/Good)				
Reasonable	0.81	(0.64; 1.00)	**0.75**	**(0.59; 0.94)**
Bad/Very bad	0.84	(0.44; 1.47)	0.76	(0.40; 1.34)
**Number of comorbidities** (ref. 0)				
1	0.91	(0.71; 1.17)	0.78	(0.60; 1.01)
≥2	**0.73**	**(0.53; 0.98)**	**0.59**	**(0.42; 0.81)**
**Have school-age children** (ref. No)	0.91	(0.71; 1.16)	1.18	(0.88; 1.57)
**Frequency of agitation, sadness or anxiety due to the physical distance measures** (ref. Never)				

Some days	**0.61**	**(0.48; 0.77)**	**0.65**	**(0.51; 0.84)**
Almost every day	0.75	(0.50; 1.09)	0.81	(0.54; 1.19)
Every day	1.13	(0.70; 1.78)	1.33	(0.81; 2.11)
**Confidence in the health services response to COVID-19** (ref. Very confident/Confident)				
Not very confident/Not confident	**2.74**	**(2.15; 3.48)**	**2.86**	**(2.23; 3.66)**
**Confidence in the health services response to non-COVID-19** (ref. Very confident/Confident)				
Not very confident/Not confident	**2.20**	**(1.77; 2.76)**	**2.27**	**(1.81; 2.85)**
**Perception of the adequacy of the measures implemented by the Government** (ref. Very adequate/Adequate)				
Not very adequate/Not adequate	**3.47**	**(2.78; 4.34)**	**4.00**	**(3.16; 5.06)**
**Self-Perceived Risk to get COVID-19 Infection** (ref. High)				
Moderate	1.22	(0.76; 2.08)	1.26	(0.78; 2.14)
Low/No risk	**2.00**	**(1.26; 3.38)**	**2.04**	**(1.27; 3.45)**
Not sure	1.40	(0.72; 2.73)	1.41	(0.72; 2.75)
**Self-Perceived Risk to Develop Severe Disease Following COVID-19 Infection** (ref. High)				
Moderate	0.85	(0.61; 1.19)	0.94	(0.67; 1.32)
Low/No risk	1.04	(0.76; 1.45)	1.26	(0.90; 1.78)
Not sure	0.77	(0.49; 1.21)	0.88	(0.55; 1.40)
**Safety perception in the COVID-19 vaccines** (ref. Totally safe/Safe)				
Not very safe/Unsafe	**13.44**	**(10.06; 18.01)**	**15.82**	**(11.67; 21.54)**
**Eficaccy perception in the COVID-19 vaccines** (ref. Completely effective/Effective)				
Not very Effective/Not effective	**8.90**	**(6.83; 11.59)**	**10.32**	**(7.81; 13.64)**

Table S2: Crude and adjusted odds of hesitancy, for participants who answered they would delay or refuse vaccination, for the determinants of vaccine hesitancy. Odds adjusted for gender, age, education and period of questionnaire. OR: odds-ratio, CI: confidence interval.

	Crude		Adjusted	
	**OR**	**95% CI**	**OR**	**95% CI**
**Gender** (ref.Male)	**0.63**	**(0.45; 0.91)**	**0.57**	**(0.40; 0.83)**
**Age** (ref.50-64)				
16-24	**3.20**	**(1.18; 7.38)**	**2.88**	**(1.05; 6.73)**
25-49	**1.81**	**(1.24; 2.67)**	**1.99**	**(1.36; 2.96)**
65-79	0.67	(0.39; 1.12)	0.63	(0.36; 1.05)
80+	1.42	(0.23; 4.88)	1.16	(0.18; 4.05)
**Education** (ref.University)				
No education/Basic education	1.13	(0.50; 2.23)	1.43	(0.62; 2.91)
Secondary	1.39	(0.94; 2.01)	1.40	(0.94; 2.06)
**Monthly household income** (ref. <650€)				
651-1000€	0.60	(0.30; 1.23)	0.62	(0.31; 1.29)
1001-1500€	**0.24**	**(0.12; 0.50)**	**0.28**	**(0.13; 0.60)**
1501-2000€	**0.51**	**(0.27; 0.99)**	**0.62**	**(0.32; 1.23)**
2001-2500€	**0.25**	**(0.12; 0.53)**	**0.31**	**(0.14; 0.68)**
> 2501€	**0.21**	**(0.11; 0.43)**	**0.29**	**(0.14; 0.62)**
**Lost of income due to the pandemic** (ref. No)	**1.68**	**(1.17; 2.37)**	**1.46**	**(1.01; 2.09)**
**Occupation** (ref. Worker)				
Student	**2.73**	**(1.17; 5.59)**	1.78	(0.58; 4.77)
Unemployed	1.11	(0.51; 2.14)	1.11	(0.51; 2.15)
Retired	**0.51**	**(0.31; 0.80)**	0.72	(0.35; 1.43)
Other	1.20	(0.60; 2.19)	1.29	(0.60; 2.51)
**Month** (ref. November)				
September	1.27	(0.79; 1.97)	1.12	(0.69; 1.76)
October	1.21	(0.83; 1.75)	1.19	(0.81; 1.73)
**Intention of taking the flu vaccine this year** (ref. Yes, I take the flu vaccine every year)				
Yes, I will take the flu vaccine this year	0.41	(0.02; 2.31)	0.5	(0.03; 2.86)
No	**20.84**	**(10.47; 49.36)**	**27.58**	**(13.02; 68.65)**
**Perception of the health status** (ref. Very good/Good)				
Reasonable	**0.45**	**(0.31; 0.65)**	**0.49**	**(0.33; 0.71)**
Bad/Very bad	0.40	(0.10; 1.08)	0.45	(0.11; 1.24)
**Number of comorbidities** (ref. 0)				
1	**0.51**	**(0.34; 0.75)**	**0.55**	**(0.36; 0.83)**
≥2	**0.14**	**(0.05; 0.28)**	**0.14**	**(0.06; 0.31)**
**Have school-age children** (ref. No)	**1.75**	**(1.24; 2.44)**	1.40	(0.95; 2.06)
**Frequency of agitation, sadness or anxiety** (ref. Never)				
Some days	**0.53**	**(0.37; 0.76)**	**0.55**	**(0.38; 0.80)**
Almost every day	**0.35**	**(0.15; 0.71)**	**0.34**	**(0.15; 0.68)**

Every day	1.12	(0.55; 2.11)	1.07	(0.51; 2.05)
**Confidence in the health services response to COVID-19** (ref. Very confident/Confident)				
Not very confident/Not confident	**9.01**	**(6.34; 12.91)**	**8.57**	**(5.99; 12.37)**
**Confidence in the health services response to non-COVID-19** (ref. Very confident/Confident)				
Not very confident/Not confident	**9.49**	**(5.92; 16.16)**	**9.32**	**(5.79; 15.92)**
**Perception of the adequacy of the measures implemented by the Government** (ref. Very adequate/Adequate)				
Not very adequate/Not adequate	**30.46**	**(18.53; 53.62)**	**29.95**	**(18.06; 53.10)**
**Self-Perceived Risk to get COVID-19 Infection** (ref. High)				
Moderate	**4.02**	**(1.23; 24.68)**	**4.19**	**(1.28; 25.83)**
Low/No risk	**8.51**	**(2.66; 51.89)**	**8.56**	**(2.66; 52.38)**
Not sure	**6.66**	**(1.73; 43.69)**	**7.51**	**(1.93; 49.50)**
**Self-Perceived Risk to Develop Severe Disease Following COVID-19 Infection** (ref. High)				
Moderate	**2.43**	**(1.16; 5.96)**	**2.29**	**(1.08; 5.63)**
Low/No risk	**4.51**	**(2.22; 10.83)**	**3.56**	**(1.72; 8.67)**
Not sure	**2.60**	**(1.05; 7.00)**	2.35	(0.94; 6.38)
**Safety perception in the COVID-19 vaccines** (ref. Totally safe/Safe)				
Not very safe/Unsafe	**121.51**	**(75.89; 202.36)**	**133.37**	**(81.40; 228.03)**
**Eficaccy perception in the COVID-19 vaccines** (ref. Completely effective/Effective)				
Not very Effective/Not effective	**58.30**	**(38.15; 91.77)**	**59.55**	**(38.45; 95.11)**
